# The role of the target language culture on Arabic learners' fondness for Arabic poetry

**DOI:** 10.3389/fpsyg.2024.1310343

**Published:** 2024-05-02

**Authors:** Li Gao, Kai Wang, Qian Yang, Yiwei Lu

**Affiliations:** ^1^Institute of Corpus Studies and Applications, Shanghai International Studies University, Shanghai, China; ^2^Department of Arabic, School of Asian and African Studies, Shanghai International Studies University, Shanghai, China

**Keywords:** fondness for Arabic poetry, Arabic learners, target language culture, EEG, frontal alpha asymmetry

## Abstract

As an important carrier of culture, poetry plays a significant role in deepening language learners' understanding of the target language culture as well as enhancing their language skills; however, the effect of the target language culture on language learners' enjoyment of poetry remains unclear. The study served as an attempt to shed light on the point of whether the target language culture has different effects on high- and low-level Chinese Arabic learners' fondness for Arabic poetry with the use of pictures related to Arabic culture and those not related to Arabic culture. In the current study, 40 Arabic learners (20 high-level and 20 low-level) scored the Arabic poem line based on their fondness for it after viewing two kinds of picture with electroencephalogram (EEG) recording. Frontal alpha asymmetry index as a correlate of approach and avoidance related motivation measured by EEG power in the alpha band (8-13 Hz) was calculated for examining whether the behavioral results of Arabic learners' fondness for poetry are in line with the results of changes in the related EEG components. Behavioral results illustrated that low-level subjects showed significantly less liking for Arabic poetry after viewing pictures related to Arabic culture compared to those not related to Arabic culture. The high-level subjects did not show a significant difference in the level of liking for Arabic poetry between the two cases. FAA results demonstrated that low-level subjects presented a significant avoidance-related responses to Arabic poetry after viewing pictures related to Arabic culture in comparison to viewing pictures not related to Arabic culture; while the FAA values did not differ significantly between the two cases in high-level subjects, which is in line with behavioral results. The findings of this research can benefit teachers in motivating students to learn poetry in foreign language curriculum and also contribute to the literature on the effect of target language culture on language learners' enjoyment of poetry.

## 1 Introduction

As one of the most sophisticated forms of literary, poetry is unquestionably an important segment of the foreign language curriculum (Maley, 1987; Finch, 2003; Cahnmann, 2006; Khan, 2020). Even though learning poetry can help foreign language learners deepen their understanding of the target language culture (Tevdovska, 2016) and improve their language skills (Mittal, 2016; Aladini and Farahbod, 2019), several studies have found that poetry learning is the least favorable literature genre among foreign language learners (Hirvela and Boyle, 1988; Vijayarajoo et al., 2019; Wai and Abidin, 2020). Since liking is one of the integrative motivations that influence language learning (Elyildirim and Ashton-Hay, 2006), there is a need to explore factors that influence learners' liking for poetry so as to motivate them to learn poetry and improve their language skills.

Factors affecting native speakers' liking for poetry have been researched in several languages, such as German poetry (Obermeier et al., 2013, 2016; Lüdtke et al., 2014; Kraxenberger and Menninghaus, 2017), English poetry (Papp-Zipernovszky et al., 2022), and haiku poetry (Hitsuwari and Nomura, 2022). However, the factors affecting foreign language learners' liking for poetry were mainly studied in English. It has been found that teaching methods (Tomlinson, 1986), language difficulty (Adeninhun, 2012), and literary beauty (Rodríguez, 2018) are significant factors that affect English learners' enjoyment of English poetry. Unlike English, which is a Western Germanic language (Bech and Walkden, 2016), Arabic is a Semitic language (Kitchen et al., 2009). Therefore, the results of English learners' liking for English poetry cannot be directly applied to the study of Arabic learners' liking for Arabic poetry because of the differences between Arabic and English. In addition, Arabic language has achieved creativity and originality in various fields of literature, the greatest of which is poetry (Dahami, 2020). As an important part of Arab heritage (Al-Shaibani et al., 2020) and the oldest art form in the Arab world, Arabic poetry has an extraordinary place in Arab culture (Kendall, 2018), and it plays a significant role in the Arabic learning process (Al-Tamimi, 2012; Rahmanian Koshkaki and Mollazadeh, 2017). However, factors influencing the Arabic learners' liking for Arabic poetry remain unknown. Thus, more attention needs to be paid to the study of Arabic learners' fondness for Arabic poetry to help them completely appreciate the beauty of the poetry and arouse their interest in learning poetry.

The reading and learning of poetry are considered to be essential for foreign language learners to understand the target language culture (Ghazali, 2016; Novasyari, 2019), which has a significant role in students' language achievement (Alptekin, 1993; Kozhevnikova, 2014). It has been found that the positive attitude of students toward the target language culture may influence or be influenced by their learning success (Tseng, 2013). In addition, several studies have discovered that positive attitudes toward the culture of the target language are conducive to improving language learning, while negative attitudes toward the target language culture may lead to low language proficiency (Oller and Perkins, 1978; Rafieyan et al., 2013), which means that students with high language achievement tend to be those who enjoy the culture of the target language and admire the speakers of that language, while the opposite is true for students with low language achievement (Falk, 1978). Although the target language culture as a vital feature of every stage of language learning has an influence on language achievements, there is still much to discover about its impact on language learners' fondness of poetry, which is an important carrier of the target language culture. Therefore, it is necessary to explore the effect of target language culture on language learners' enjoyment of poetry and to further study whether this effect differs across language achievements.

Since there is still a lack of evidence on whether and how the target language culture influences Arabic learners' enjoyment of Arabic poetry, a neuroscience experiment has been conducted in the current study to explore whether target language culture has an effect on Arabic learners' fondness for Arabic poetry, and if so, whether this effect varies between high- and low- level Arabic learners. Given that pictures as a rich source in foreign language classrooms (Tuttle, 1975) can help increase language learners' learning motivation and develop a mental image of the target culture (Wright, 1989; Peck, 1998; Bush, 2007; Klasone, 2013; Philominraj et al., 2017), the pictures related to Arab culture and the pictures unrelated to Arab culture are both used in the neuroscience experiment.

Neurocognitive poetics is defined as “the transdisciplinary empirical investigation of and theorizing about (poetic) literature reception by eye or ear including its neuronal underpinnings (Jacobs, 2015).” Some studies have introduced the idea of drawing upon neuroscience tools in studying students' poetry reading process (Fechino et al., 2020; Cartocci et al., 2021). There are several advantages in studying whether and how the target language culture will influence Arabic learners' enjoyment of Arabic poetry by conducting a neuroscience experiment. First, neuroscience experiments can contribute to unraveling the neural basis of cognition and emotion, providing new insights into the impact of target language culture on Arabic learning. Second, neuroscience tools help reveal hidden mental processes among students while reading poetry to provide a new insight into the differences in students' enjoyment of poetry, which can help teachers have a better understanding of the role of target language culture in Arabic learners' fondness for Arabic poetry.

To the best of our knowledge, the current exploratory study is the first to preliminarily explore the impact of target language culture on Arabic learners' enjoyment of Arabic poetry from the perspective of neurocognitive poetics, and it contributes to the research on target language culture and poetry enjoyment in foreign language learning. In addition, the findings of the study will provide teachers with some insights into customizing strategies and methods for teaching poetry to foreign language learners.

## 2 Literature review

### 2.1 Target language culture and language learning

Given the interconnectedness of language and culture, language learning is not only about learning the language itself but also about learning the culture of that language (Pourkalhor and Esfandiari, 2017). Previous studies have shown that learning the culture of the target language is conducive to stimulating language learners' interest in learning and improving their language proficiency. For instance, Alrasheedi (2020) discovered that students' lack of knowledge of the culture related to the target language is one of the difficulties in developing their speaking skills. In addition, it has been suggested that introducing English language learners to the culture of the target language helps improve their sociocultural competence, which will lead to higher levels of English proficiency (Khouni and Boudjelal, 2019). Setiadi et al. (2022) further found that incorporating target culture into English language teaching materials has the potential to increase students' interest in English, which in turn improves their motivation to learn English.

However, although learning the target language culture can be an effective method for improving students' foreign language proficiency, language learners are not passive recipients of the target language culture, and their perceptions of the target language culture can also have an impact on their attitudes toward learning foreign language, which in turn affects their language proficiency (Getie, 2020; Orfan, 2020). More specifically, positive attitudes toward the target language culture can help language learners make more progress in the foreign language learning process, while negative attitudes toward the target language culture are not conducive to improving their language proficiency (Prastiwi, 2018). Therefore, the influence of target language culture on foreign language proficiency is not simply a matter of taking the content of the target language culture as a part of the foreign language learning content to help students improve their language proficiency naturally. How students perceive the content related to the target language culture is also an important link that cannot be ignored in foreign language learning and teaching.

Given that the study of poetry is an important element in the foreign language learning classroom (Khan, 2020; Kilag et al., 2023), the relationship between the study of poetry and the culture of the target language has also received academic attention. It has been found that the study of poetry can help language learners understand the target culture more deeply and thus further improve their language proficiency (Tevdovska, 2016; Zengin et al., 2019; Ali et al., 2021). Since the existing studies have mainly focused on the effect of poetry learning on students' understanding of the target culture, the effect of the target culture on foreign language learners' poetry learning, especially their enjoyment of poetry, is still in its infancy. In addition, prior research has focused mainly on English language learning, with little coverage of Arabic, which is a language family different from that of English (Akan et al., 2019) and the sixth most widely spoken language in the world (Farghaly, 2010). Moreover, previous studies have used questionnaires to ask participants to subjectively rate their liking for poetry (Koehler et al., 2005; Wang et al., 2021), but they have not studied the subjects' neural components when they are reading poetry. Thus, there is still a lack of empirical research on how the target culture affects Arabic learners' enjoyment of poetry and whether this effect varies according to students' level of Arabic language proficiency. To address this issue, the present study explored the effect of target culture on Arabic learners' enjoyment of Arabic poetry and its underlying neural basis through a combination of electroencephalography (EEG) recordings and online scoring.

### 2.2 Approach-avoidance motivation and FAA

Electroencephalography (EEG) has uncovered that the frontal alpha asymmetry (FAA), measured by EEG power in the alpha band (8–13 Hz), is commonly used as an indicator of action motivation (approach vs. avoidance; Davidson et al., 1990; Briesemeister et al., 2013; Cartocci et al., 2018; Goldstein et al., 2021). More precisely, approach-related motivation and tendency to appreciate stimuli are correlated with relatively more activity in the left lateral prefrontal cortex, whereas withdrawal-related motivation and tendency to avoid stimuli are associated with relatively more activity in the right lateral prefrontal cortex (Berkman and Lieberman, 2010; Schneider et al., 2016; Silva-Passadouro et al., 2022).

Several studies have drawn on the FAA to understand action motivation in the aesthetic process of different art forms like sculpture (Babiloni et al., 2014), painting (Maglione et al., 2017), and music (Cartocci et al., 2018). In addition, researchers have begun to analyze subjects' responses to different literary materials with the help of the FAA index and have confirmed that FAA provides a reliable means that can be used to distinguish between approach motivation and avoidance motivation (Brouwer et al., 2015; Cartocci et al., 2021). Although poetry as an important literary form has been addressed in studies in this field, the existing studies have been limited to auditory poetry stimuli (Cartocci et al., 2021) and have not addressed visual poetry stimuli, especially the reading of Arabic poetry, which is an essential part of the Arab cultural heritage (Al-Shaibani et al., 2020). Thus, in order to fill this gap, this study drew upon the results of FAA to understand the behavioral motivations of Arabic learners in reading Arabic poetry.

Several equations have been used in the literature to calculate FAA, such as subtracting the log-transformed absolute alpha power of the left frontal region (F3) from the analogous log-transformed alpha power of the right frontal region (F4) (Davidson, 1992; Cheung et al., 2019); subtracting the natural log of the alpha power of a frontal electrode in the left hemisphere (F3) from that of the homologous right frontal electrode (F4) and dividing this difference score by the addition of left and right frontal alpha power (Mathersul et al., 2008); and subtracting the alpha power of the left frontal region (F3) from the alpha power of the right frontal region (F4) and dividing this difference score by its sum (Van Der Vinne et al., 2017). In our study, the last equation was used because it is the closest to the normal distribution (Arns et al., 2016). Positive alpha asymmetry indices denote greater relative left frontal activity, which is associated with approach-oriented behaviors, while negative alpha asymmetry indices denote greater relative right frontal activity, which is consistently linked with avoidance-related motivation (Horan et al., 2014; Lagast et al., 2020; Beik et al., 2022; Silva-Passadouro et al., 2022); hence, there is substantial evidence that the frontal alpha asymmetry is a valid index for action motivation (Fischer et al., 2018; Takehara et al., 2020; Anaya et al., 2021). Therefore, we chose to calculate the FAA index to verify whether the behavioral results of Arabic learners' fondness for poetry are consistent with the results of changes in the EEG components associated with action motivation when Arabic learners read Arabic poetry after viewing pictures related and unrelated to Arab culture.

### 2.3 Hypothesis

On the basis of previous findings that high-level language learners tend to like the culture of the target language, it is hypothesized that high-level Arabic learners show approach-oriented behaviors toward pictures related to Arabic culture compared to pictures unrelated to Arabic culture, thus contributing to the emergence of an increase in their liking of Arabic poetry. In contrast, low-level language learners tend to have more negative attitudes toward the target language culture; thus, it is hypothesized that low-achievement Arabic learners show avoidance-oriented behaviors toward pictures related to Arabic culture compared to those un related to Arabic culture, which will lead to a decrease in their enjoyment of Arabic poetry. Moreover, when the subjects read poetry after viewing pictures related to Arab culture, the FAA values at high-level Arabic learners are hypothesized to be more positive when compared to reading poetry after viewing pictures unrelated to Arab culture, whereas when the subjects read Arabic poetry after viewing pictures related to Arab culture, the FAA index of low-level Arabic learners is more negative when compared to reading Arabic poetry after viewing pictures unrelated to Arab culture, which is in line with behavioral results.

## 3 Materials and methods

### 3.1 Participants

A total of 40 healthy university students majoring in Arabic (19 women and 21 men), with a mean age (19–29 years) of 22.28 years and S.D. = 2.124, were recruited as participants from Shanghai International Studies University, a Chinese public university that specializes in foreign languages, international relations, and translation studies. All of the participants are native Chinese speakers learning Arabic as their third language. They are right-handed, have normal or corrected-to-normal vision, and have no reading impairment, and none of them have any history of neurological diseases or substance abuse. They were divided into two groups according to their Arabic proficiency level before the formal experiment, each with 20 subjects. The high-level proficiency group consisted of 10 female and 10 male subjects, with a mean age (19–29 years) of 22.95 years, S.D. = 2.373. The low-level proficiency group consisted of nine female and 11 male subjects, with a mean age (19–25 years) of 21.6 years, S.D. = 1.635. They signed a written informed consent before the formal experiment. In addition, the high-level proficiency group included not only undergraduate students but also graduate students, which makes the language levels of the two groups differ in some ways. Despite China's large population, the total number of colleges and universities in China that offer graduate and undergraduate programs related to the Arabic language is relatively small. According to the data from the China Graduate Admissions Information Network, the total number of Chinese universities that offer both graduate and undergraduate majors in Arabic, including Shanghai International Studies University (SISU), is 17, of which SISU enrolls fewer than 25 graduate students in Arabic-related majors each year. Moreover, taking into account the balanced gender ratio of male and female subjects, 40 university students majoring in Arabic (19 female and 21 male subjects) are sufficient to validate the results.

The experiment was approved by the Internal Review Committee of Shanghai International Studies University. All the procedures performed in this study complied with the ethical standards of the Institutional and National Research Committee and with the 1964 Helsinki Declaration and its later amendments or comparable ethical standards (World Medical Association, 2013).

### 3.2 Materials

The hypotheses proposed in this study were examined through a 2 × 2 mixed-design experiment (two levels of Arabic language proficiency (between-subject) and two kinds of priming pictures (within-subject). The whole experiment consisted of 180 stimuli, which consisted of two kinds of priming picture stimuli (45 Arab-related pictures and 45 non-Arab-related pictures) and 90 poem stimuli. Each stimulus was presented once, with two stimuli per trial (a picture stimulus and a poem stimulus), for a total of 90 trials.

We manipulated the Arabic language proficiency by dividing the subjects into two groups (high-level and low-level) based on their learning hours in Arabic, their teachers' evaluation of their classroom performance, and their scores in the Arabic IV exam. To confirm the statistical significance of this division, we had them take a test developed by a team of five experts (three Chinese experts in Arabic and two native Arabic-speaking foreign teachers). The test consisted of 24 multiple-choice questions (one point for each) and covered multiple aspects such as reading comprehension, grammar selection, and vocabulary discrimination. Test scores of each subject are listed in the Appendix. An independent sample *t*-test was computed by SPSS-26 to compare the test scores between the two groups, and the results (as shown in Table 1) showed a significant difference (*p* < 0.001).

**Table 1:** *T*-test results for Arabic test scores between two subject groups.

**Group**	**No**.	**Mean**	**St. deviation**	***t*-value**	**Sig. (2-tailed)**
High-level	20	19.85	2.581	4.759	*p* < 0.001
Low-level	20	13.60	5.276		

The two kinds of priming pictures were manipulated by using pictures with two kinds of content (90 pictures in total, 45 Arab-related pictures and 45 non-Arab-related pictures). These materials were selected from a pilot pool of pictures including 60 Arab-related pictures and 60 non-Arab-related pictures of similar sizes and clarity sourced through search results of Google Pictures. The Arab-related pictures depict urban landscapes and natural scenery in the Arab world and convey a distinct impression of Arab culture, while the non-Arab-related pictures are not associated with the Arab world and do not depict elements of Arab culture or geography. A total of 15 students and faculty of Arabic language majors who were unaware of the experiment were tasked with rating these pictures on a 1–7 scale (1 = strongly disagree; 7 = strongly agree) regarding the pictures' relation to Arabic culture and their ability to remind them of Arabic culture. The mean scores of the two questions were then calculated. The 45 pictures with the highest scores (most culturally relevant to the Arab world) and the 45 pictures with the lowest scores (least culturally relevant to the Arab world) were selected as the formal experimental materials, ensuring a contrast between the two types of pictures. A *t*-test of the scores for these 90 pictures showed a significant difference between the two styles (*p* < 0.001), as shown in Table 2.

**Table 2:** *T*-test results for scores of two picture styles.

**Group**	**No**.	**Mean**	**St. deviation**	***t*-value**	**Sig. (2-tailed)**
Arab-related pictures	45	6.292	0.242	85.449	*p* < 0.001
Non-Arab-related pictures	45	2.537	0.168		

As for the poem stimuli in the experiment, a collection of 120 lines of poetry written by the well-known contemporary poet Adonis was compiled in the pilot phase of the study. These poem lines were reviewed by three Chinese experts in Arabic and two Arabic foreign teachers, who scored them based on their comprehensibility and rhyme. The top 90 lines with the highest scores were chosen as the formal experimental materials to ensure that the vocabulary and expressions in these materials could be understood by all subjects.

### 3.3 Procedure

The experiment was conducted in a room with electrical isolation, in which participants were seated in front of a computer screen. The experimental stimuli were presented on the screen, located 100 cm from the participants. The E-prime 3.0 software (Psychology Software Tools, Pittsburgh, PA) was used to present the stimuli, record responses, and transmit triggers. Participants responded to the stimuli using a keypad. The experiment consisted of 90 trials in total, which were divided into six blocks of 15 trials each. The order of presentation of trials and the combination of picture and poem stimuli within each trial were randomized. Three practice trials were administered before the formal experiment.

**Figure 1:**
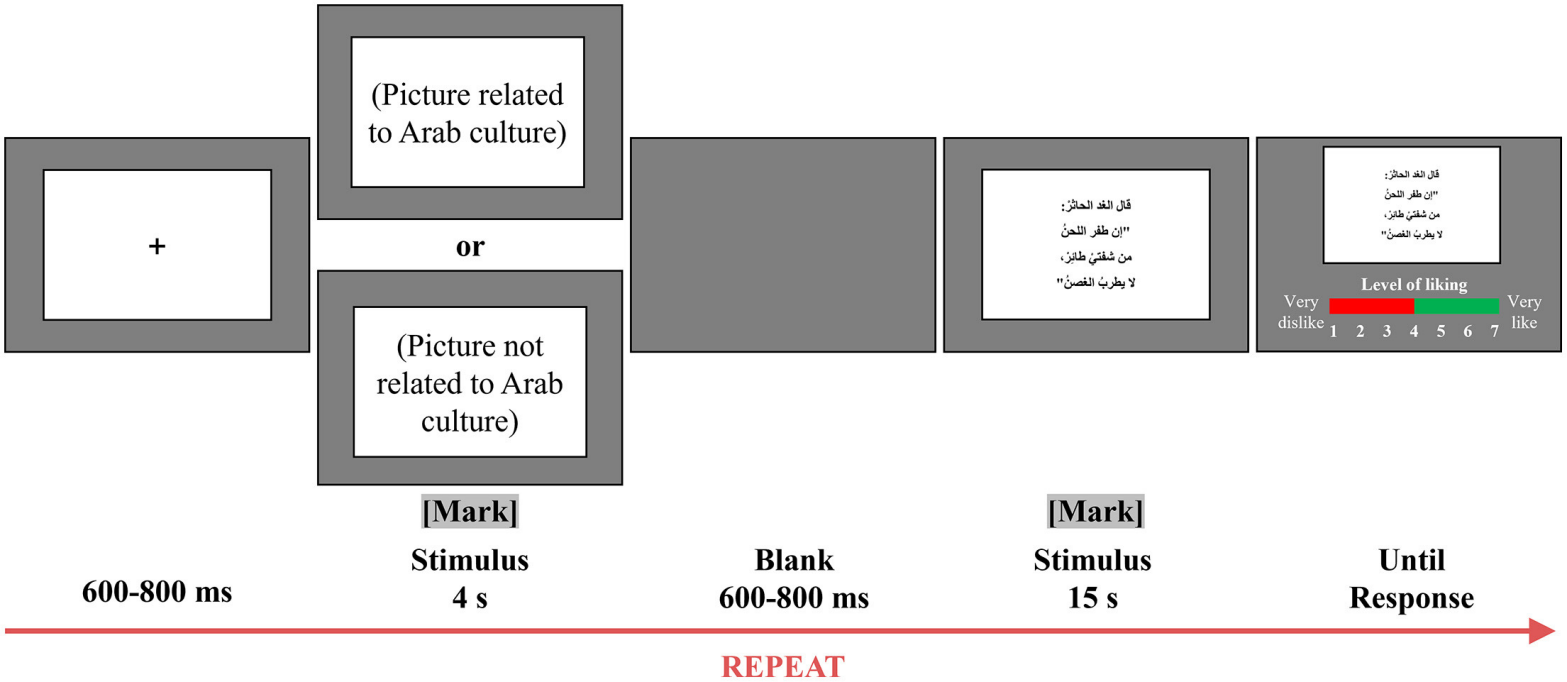
A single trial in the evaluation task for personal preference of poem lines. Each trial began with the presentation of a fixation cross. Participants then viewed a randomly presented image (either culturally relevant to the Arab world or not) for 4 s. After a blank screen interval of 600–800 ms, the screen displaying the poem line was presented for 15 s. Participants were then asked to indicate the extent to which they liked the poem line by pressing the corresponding button. EEG recordings were taken throughout the experiment.

A single trial of the experiment is depicted in Figure 1. Each trial began with a fixation display presented on a black screen for a duration of 600–800 ms. Subsequently, a random image (either Arab-related or non-Arab-related) was presented for 4 s and marked for EEG analysis. After a 600–800-ms interval, the page displaying the Arabic poem lines was automatically displayed for 15 s and also marked for EEG analysis. Participants were then asked to score the poem line they had just read on a 7-point scale (1 = very dislike and 7 = very like) based on their fondness for it, with key 1 indicating a decrease in the level of liking, key 2 indicating confirmation, and key 3 indicating an increase in the level of liking.

### 3.4 EEG recording and data analysis

EEG data were recorded from 32 scalp sites using the Brain Product (Gilching, Germany) system according to the 10–20 System of Electrode Placement. The impedances of all scalp electrodes were <10 kΩ, and the sampling rate was set at 500 Hz. The FCz served as the online reference electrode.

The preprocessing of the picture stimuli EEG data and the poem stimuli EEG data was performed with the same progress, separately. We preprocessed the offline data using Toolbox EEGLAB 12.0.2 (Delorme and Makeig, 2004) and in-house MATLAB scripts. The EEG data were first re-referenced to the average of the two mastoids. They were then filtered with a low-pass filter of 30 Hz and a high-pass filter of 0.1 Hz. EEG epochs were segmented from −1,000 to +4,000 ms (picture stimuli) and −1,000 to +15,000 ms (poem stimuli) around the stimulus onsets. An independent component analysis (ICA) was applied to reject artifacts caused by eye blinks (de Bruijn et al., 2020). Technical and other types of noise were also eliminated if the EEG amplitudes exceeded ±100 μV. The data were then baseline-corrected (200 ms before picture onset).

For behavioral data, we analyzed scores given by subjects to the poem lines after two picture priming types using a 2 × 2 repeated measures ANOVA. The 2 × 2 design included two levels of subject proficiency (high-level vs. low-level) and two picture priming types (Arab-related vs. non-Arab-related).

For EEG data, we analyzed the frontal alpha asymmetry (FAA) of the preprocessed EEG data using a 2 × 2 repeated measures ANOVA for both picture and poem stimuli. To calculate the FAA value for picture stimuli, we applied a short-time Fourier transform (STFT) to the preprocessed EEG data and extracted data from the F3 and F4 electrodes in the alpha frequency band (8–13 Hz) between 550 and 1,150 ms after stimulus onset. We then used the formula [right alpha (F4) – left alpha (F3)]/[right alpha (F4) + left alpha (F3)] to calculate the FAA, without log-transforming the data to better approximate a normal distribution (Arns et al., 2016). We followed the same procedure for poem stimuli, except that we first divided the EEG epoch of the poem stimuli (0–15,000 ms) into five equal segments of 3,000 ms each and averaged them to obtain a 0–3,000-ms epoch. We then computed the FAA for poem stimuli from the new epoch's data at the F3 and F4 electrodes in the alpha frequency band (8–13 Hz) between 1,200 and 1,250 ms after stimulus onset.

## 4 Results

### 4.1 Behavioral results

**Figure 2:**
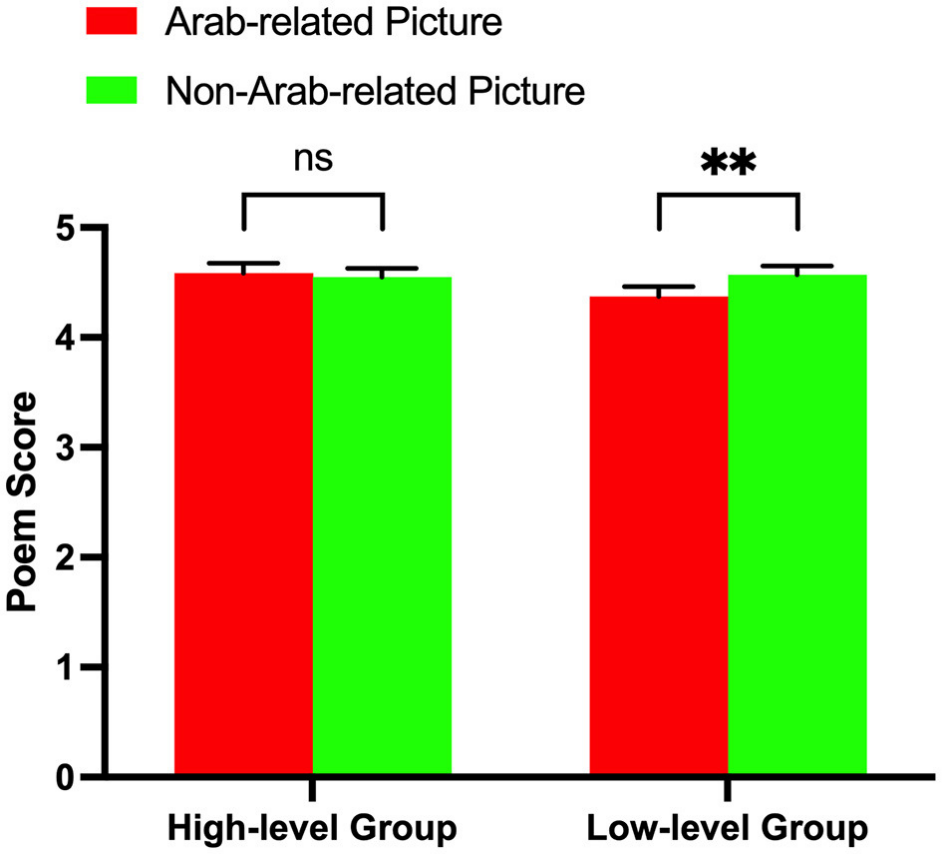
Poem scores of two subject groups after two picture priming types. The simple effect analysis of behavioral results showed a significant difference in the low-level proficiency group between poem scores after Arab-related picture priming and those after non-Arab-related picture priming, while the difference was not significant in the high-level group. **p < 0.01. ns, not significant.

The results of the two-way 2 (subject proficiency levels: high vs. low) × 2 (picture priming types: Arab-related pictures vs. non-Arab-related pictures) repeated measure ANOVA indicated no main effect for subject proficiency level [*F*_(1, 38)_ = 0.746, *p* = 0.393, η^2^ = 0.019] and no main effect for picture priming type [*F*_(1, 38)_ = 2.601, *p* = 0.115, η^2^ = 0.064]. However, there was a significant interaction effect between subject proficiency level and picture priming type [*F*_(1, 38)_ = 5.442, *p* = 0.025, η^2^ = 0.125]. A further simple effect analysis indicated that the difference between poem scores after Arab-related picture priming and those after non-Arab-related picture priming was significant for low-level Arabic learners [*F*_(1, 38)_ = 7.784, *p* = 0.008, η^2^ = 0.170], which indicated that low-level Arabic learners had scored poem lines significantly higher after viewing non-Arab-related pictures (M = 4.568 points, S.E. = 0.080) than after viewing Arab-related pictures (M = 4.373 points, S.E. = 0.090). However, this difference was not significant for high-level Arabic learners [*F*_(1, 38)_ = 0.259, *p* = 0.614, η^2^ = 0.007]. The results are shown in Figure 2.

### 4.2 FAA results of picture stimuli

**Figure 3:**
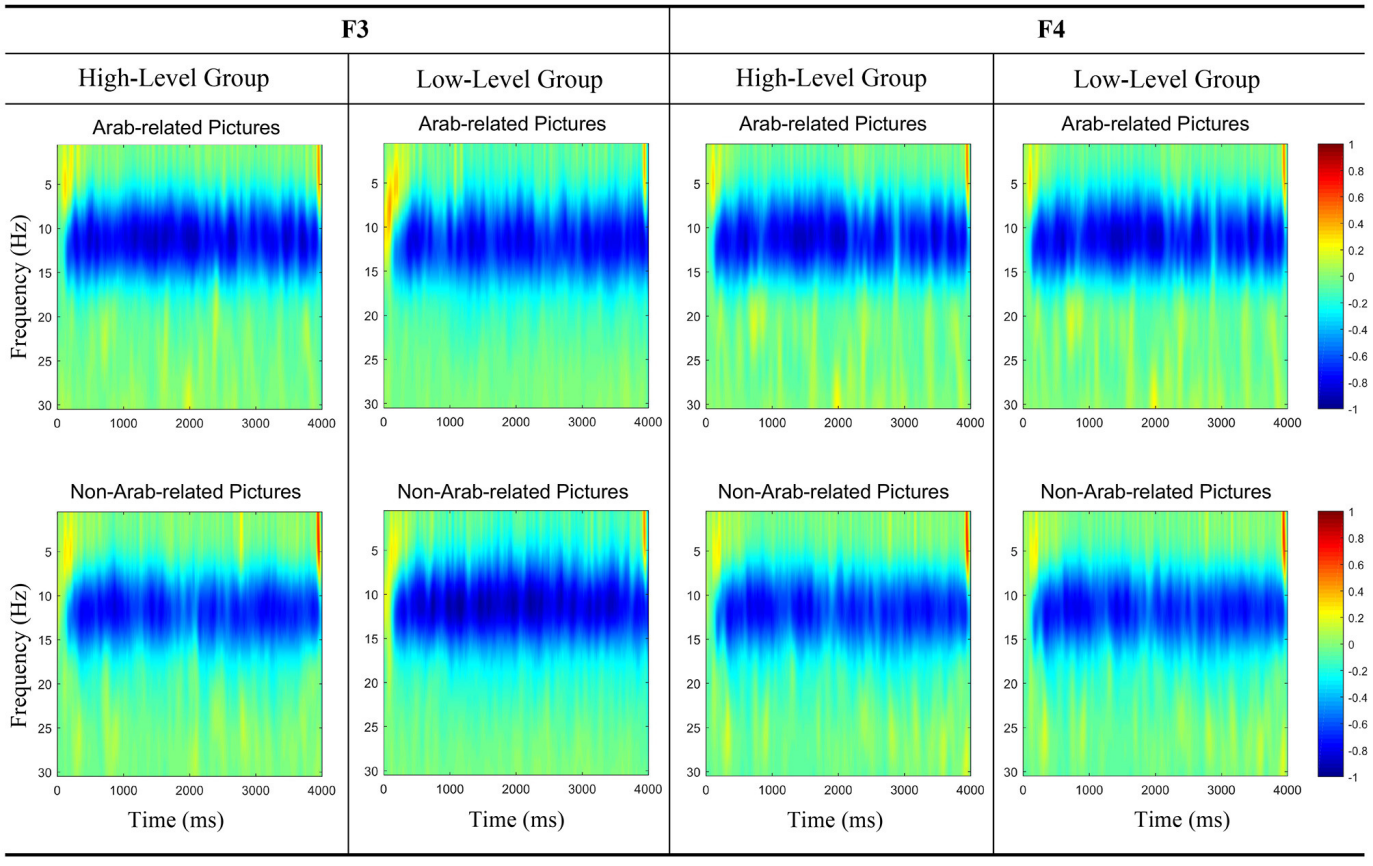
Illustration of alpha-band oscillations in F3 and F4 for Arab-related pictures and non-Arab-related pictures between the high-level proficiency group and the low-level proficiency group.

**Figure 4:**
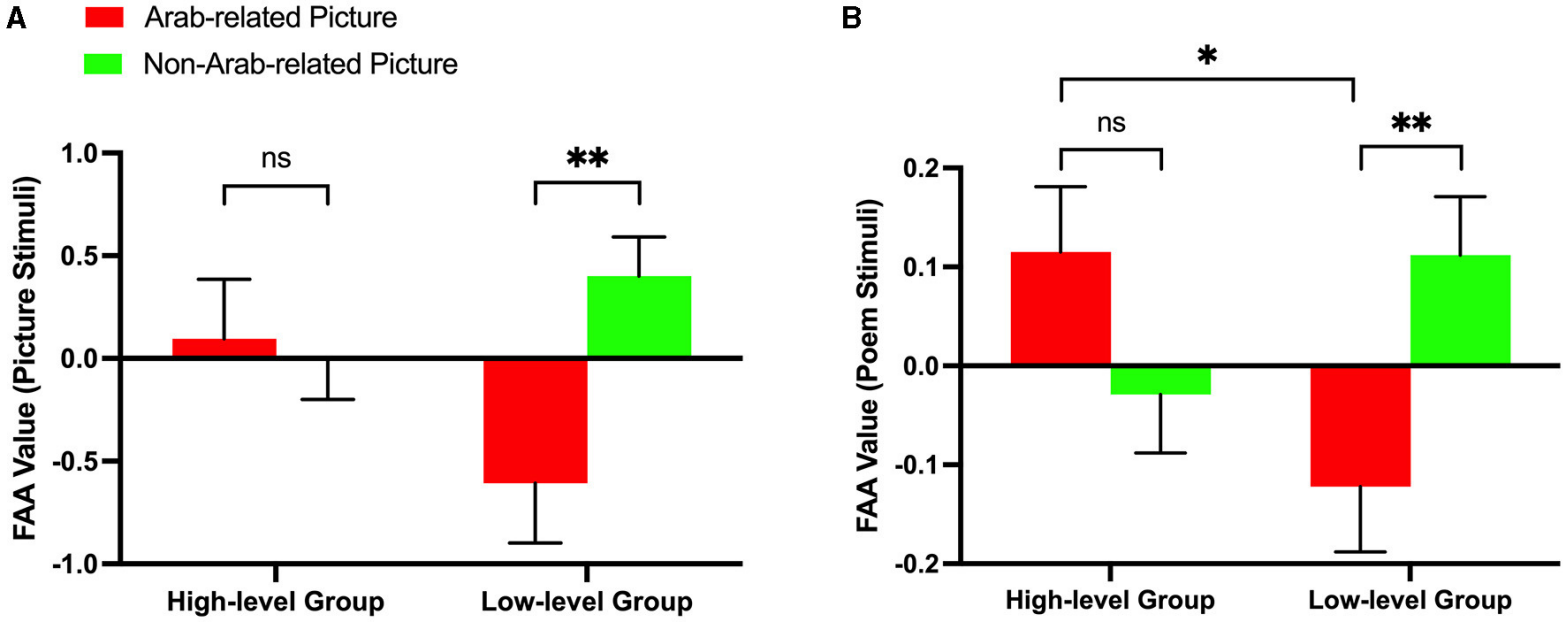
EEG data results of picture stimuli and poem stimuli. **(A)** Bar graph of FAA results for picture stimuli. **(B)** Bar graph of FAA simple analysis results for poem stimuli. Error bars indicate standard error across participants. Error bars indicate standard error across participants. *p < 0.05; **p < 0.01. ns, not significant.

The results of the two-way 2 (subject proficiency levels: high vs. low) × 2 (picture types: Arab-related vs. non-Arab-related) repeated measure ANOVA of picture stimuli FAA showed no significant main effect for subject proficiency level [*F*_(1, 38)_ = 3.530, *p* = 0.525, η^2^ = 0.085] and no significant main effect for picture type [*F*_(1, 38)_ = 1.582, *p* = 0.089, η^2^ = 0.040] but indicated a significant interaction effect between subject proficiency level and picture style [*F*_(1, 38)_ = 8.392, *p* = 0.038, η^2^ = 0.181]. The further simple effect tests elicited a significant difference in the FAA value between Arab-related pictures and non-Arab-related pictures in the low-level group [*F*_(1, 38)_ = 7.572, *p* = 0.009, η^2^ = 0.166], which is consistent with the behavioral results; however, the tests elicited no significant difference in the high-level group [*F*_(1, 38)_ = 0.081, *p* = 0.777, η^2^ = 0.002]. This result suggests that low-level subjects showed avoidance-related motivation when viewing Arab-related pictures (M = −0.608, S.E. = 0.290) compared to when viewing non-Arab-related pictures (M = 0.401, S.E. = 0.191), while high-level subjects did not experience a significant difference in action motivation when viewing either type of the picture (Arab-related picture: M = 0.096, S.E. = 0.290; non-Arab-related picture: M = −0.008, S.E. = 0.191). The ANOVA results are presented in Figure 4A and the relevant brain activity in Figure 3.

### 4.3 FAA results of poem stimuli

The results of the two-way 2 (subject proficiency levels: high vs. low) × 2 (picture priming types: Arab-related pictures vs. non-Arab-related pictures) repeated measure ANOVA of poem stimuli FAA showed no significant main effect of subject proficiency level [*F*_(1, 38)_ = 0.468, *p* = 0.498, η^2^ = 0.012] and no significant main effect of picture priming type [*F*_(1, 38)_ = 0.708, *p* = 0.405, η^2^ = 0.018]. However, we observed a significant interaction effect between subject proficiency level and picture priming type [*F*_(1, 38)_ = 12.669, *p* = 0.001, η^2^ = 0.250], which is in line with the previous behavioral results and picture stimuli FAA results. The further simple effect analysis showed that the low-level proficiency subjects showed significantly different action motivations [*F*_(1, 38)_ = 9.684, *p* = 0.004, η^2^ = 0.203] when reading poem lines after viewing Arab-related pictures (M = −0.122, S.E. = 0.066) compared to when reading them after viewing non-Arab-related pictures (M = 0.112, S.E. = 0.059), while high-level subjects did not experience a significant difference in action motivations [*F*_(1, 38)_ = 3.693, *p* = 0.062, η^2^ = 0.089] when reading poem lines after viewing either type of pictures (Arab-related picture: M = 0.115, S.E. = 0.066; non-Arab-related picture: M = −0.029, S.E. = 0.059). Additionally, there was a significant difference in FAA values between high-level subjects and low-level subjects when reading poem lines after viewing Arab-related pictures [*F*_(1, 38)_ = 6.571, *p* = 0.014, η^2^ = 0.147], compared to non-Arab-related pictures [*F*_(1, 38)_ = 2.861, *p* = 0.099, η^2^ = 0.070]. This finding suggests that, when reading poem lines, the action motivations between high-level and low-level subjects were significantly different, especially in the case of Arab-related pictures. The ANOVA results are depicted in Figure 4B.

## 5 Discussion

This study set out to explore whether the target language culture has different effects on the fondness of high- and low-level Chinese Arabic learners for Arabic poetry with the use of pictures related to Arabic culture and those unrelated to Arabic culture. For this purpose, participants were divided into high- and low-level groups based on their Arabic language proficiency. Both groups scored the Arabic poem line according to their liking for it after viewing pictures related to Arab culture, which can create a sense of context for Arabic culture, and pictures unrelated to Arab culture, which can create a sense of context for non-Arabic culture. The results of the study demonstrated that there was no significant difference in the effect of the two different pictures on high-level Arabic learners' liking for Arabic poetry, which is inconsistent with the hypothesis. Conversely, the two styles of pictures induced different action motivations in low-level subjects and evidently affected the low-level subjects' fondness for Arabic poetry, with a significant decrease in likeness scores following pictures related to Arab culture that induced avoidance motivation in low-level subjects compared to pictures unrelated to Arab culture that provoked approach motivation in them, which is in line with our proposed hypotheses. The present empirical evidence showed that differences in picture style have a significant effect only on low-level Arabic language learners' liking for Arabic poetry and that low-fluency Arabic learners' action motivation is positively correlated with their liking for Arabic poetry, which can be observed by the FAA index.

In terms of the behavioral results, high-level Arabic learners rated their liking of Arabic poetry higher on the mean score after viewing Arabic culture-related picture styles than after viewing non-Arab culture-related pictures, yet there was no significant difference in the scores between the two cases. In contrast, there was a notable discrepancy in the influence of two kinds of pictures on low-level subjects, i.e., low-level subjects were less likely to enjoy Arabic poetry after viewing pictures related to Arab culture compared to viewing pictures unrelated to Arab culture. As outlined in the introduction, high-level learners may have more positive attitudes toward the target language culture, and the opposite is true for low-level learners (Falk, 1978; Tseng, 2013). However, our study showed that high-level Arabic learners' enjoyment of Arabic poetry did not increase significantly by viewing pictures related to Arabic culture, which is not in line with our hypothesis, while low-level Arabic learners' likeness of Arabic poetry decreased significantly in the same situation, which is consistent with our hypothesis.

Analyses of subjects' action motivation while viewing the picture and poetry stimulus material by neuroscientific methods, as reflected in the FAA, also supported behavioral results. The negative FAA index was observed when the low-level subjects read Arabic poems after viewing pictures related to Arab culture, whereas a positive FAA index was found when they read Arabic poetry after viewing pictures unrelated to Arab culture. In addition, there was no significant difference in the FAA index between the two conditions when high-level subjects read Arabic poetry after viewing pictures related or unrelated to Arabic culture. As pointed out in the literature review, the frontal alpha asymmetry is a measure of the subjects' action motivation (approach vs. avoidance; Goldstein et al., 2021; Silva-Passadouro et al., 2022). Positive FAA reflects a subjective approach behavior, while negative FAA represents a subjective avoidance behavior (Al-Ezzi et al., 2020; Beik et al., 2022). Therefore, the results of FAA lend support to our hypothesis that low-fluency Arabic learners show avoidance-oriented behaviors toward pictures related to Arabic culture compared to those unrelated to Arabic culture. In addition, to verify that the change in subjects' action motivation while viewing Arabic poem lines was caused by the pictures, we also calculated the FAA values on the picture stimuli and found that the FAA results were consistent with the FAA results on poetry stimuli. This finding supported our hypothesis that the action motivations experienced by low-level subjects when reading Arabic poem line after viewing pictures related and unrelated to Arab culture are closely associated with the pictures themselves.

Both behavioral and EEG results showed that pictures related to Arab culture induced strong avoidance motivation in low-level subjects compared to pictures unrelated to Arab culture and therefore lowered their fondness for Arabic poetry, while high-level subjects did not have significant differences in their liking for Arabic poetry in the two cases. Additionally, this study pointed out the key role of pictures in Arabic language learners' fondness for Arabic poetry. Our study revealed that, compared to high-level language learners, low-level language learners' liking for Arabic poetry was more likely to be influenced by different kinds of pictures.

The differences shown by high- and low-level subjects in the influence of the two kinds of pictures on their liking for poetry may be attributed to the differences in the Foreign Language Anxiety (FLA) experienced by language learners in language acquisition. Several studies have demonstrated that anxiety plays a vital part in students' learning of Arabic language (Hasan and Al-Hasani, 2019; Mokhtar and Haron, 2021) and revealed a negative correlation between FLA and students' performance and achievement in learning Arabic (Khaldieh, 2000; Elkhafaifi, 2005; Mokhtar, 2020). This finding indicates that Arabic learners with low achievement experience more anxiety compared with Arabic learners with high achievement. Therefore, in combination with the results of the FAA, it was found that pictures related to Arabic culture that can create a sense of Arabic context may induce foreign language anxiety in low-level subjects compared to pictures unrelated to Arabic culture, which may cause them to show significant avoidance behaviors toward pictures related to Arabic culture and significant approach behaviors toward pictures unrelated to Arabic culture, which ultimately led to significant differences in the level of fondness for Arabic poetry. Compared to low-level Arabic learners, pictures related to Arabic culture did not induce significant foreign language anxiety in high-level Arabic learners, so there was no significant difference in behavioral motivation when viewing the two types of pictures, which resulted in no significant change in their enjoyment of Arabic poetry.

The current study may have several pivotal implications. First, although some research studies have explored the importance of poetry in helping language learners gain an insight into the culture of the target language (Celik and Yildiz, 2019; Zengin et al., 2019; Ali et al., 2021), limited research has been conducted on the impact of the target language culture on language learners' enjoyment of poetry, and it is unclear whether there are differences in its effect on language learners at different levels. Therefore, the results of our study have notable theoretical significance for recent advancements in this area. Second, frontal alpha asymmetry as a neural phenomenon has been studied in relation to a fondness for different art forms, such as painting (Vartanian and Goel, 2004), movies (Zhao et al., 2018), and music (Naser and Saha, 2021), but has not yet been addressed in terms of its relationship to poetry enjoyment. Our study found that frontal alpha asymmetry was highly correlated with language learners' enjoyment of poetry, thus filling the gap in this area to some extent. Third, pictures as one of the important visual materials in foreign language classrooms (Tuttle, 1975; Hafidz Zaid, 2020; Kumar, 2021) play a part in language learning (Bush, 2007; Pratiwi and Ayu, 2020; Karya et al., 2022). Based on previous findings, the current study has shown that the effect caused by different styles of pictures is related to language learner's proficiency level, which is in line with the results of existing studies examining the effect of pictures on language learning. Fourth, in terms of practical significance, the results can be used in teaching Arabic learners to learn Arabic poetry. For example, since the language level of Arabic beginners is generally low, pictures can be used in teaching to help motivate beginners to learn poetry and thus improve their language level, while more care should be taken in the choice of pictures to avoid negatively affecting the learning of poetry for low-level students.

There are two major limitations in this study that could be addressed in future research. First, both classical Arabic poetry and modern Arabic poetry hold an important place in Arabic literature (Haydar, 1981; Arifianto, 2020; Abandah et al., 2022). Moreover, in addition to modern Arabic poetry, the study of classical Arabic is also an essential part of Arabic language learning (Osman et al., 2022). Only modern Arabic poetry was studied in this research; thus, whether the results can be extended to classical Arabic poetry remains unanswered. The other limitation is in relation to the selection of target language culture-related stimulus materials. In our study, only pictures related and unrelated to the target language culture were selected as stimulus materials, and the effect of other stimulus materials related to the culture of the target language such as video and audio on language learners' enjoyment of poetry remains to be explored.

## 6 Conclusion

This study explores the impact of target language culture on Arabic language learners' fondness for Arabic poetry and whether this effect varies according to students' level of Arabic language proficiency with a 2-level Arabic language level (low vs. high) × 2-kind of picture (Arabic culture-related vs. Arabic culture-unrelated) experimental design by utilizing electroencephalogram (EEG) recordings. Both behavioral and EEG results illustrated that low-fluency subjects showed avoidance-oriented behaviors toward pictures related to Arab culture compared to those unrelated to Arab culture and therefore lowered their fondness for Arabic poetry, while high-level subjects do not have significant differences in their liking for Arabic poetry in the two cases. The findings of this research contribute to the literature on the effect of target language culture on language learners' enjoyment of poetry and provide teachers with some references for customizing strategies and methods for teaching poetry to foreign language learners.

## Data availability statement

The original contributions presented in the study are included in the article/supplementary material, further inquiries can be directed to the corresponding author.

## Ethics statement

The studies involving humans were approved by Ethics Committee of Shanghai Key Laboratory of Brain-Machine Intelligence for Information Behavior, Shanghai International Studies University, Shanghai, China (CN). The studies were conducted in accordance with the local legislation and institutional requirements. The participants provided their written informed consent to participate in this study.

## Author contributions

LG: Conceptualization, Formal analysis, Investigation, Methodology, Project administration, Software, Supervision, Validation, Writing – original draft, Writing – review & editing. KW: Formal analysis, Methodology, Software, Validation, Visualization, Writing – original draft. QY: Formal analysis, Investigation, Methodology, Writing – original draft. YL: Conceptualization, Funding acquisition, Investigation, Methodology, Project administration, Supervision, Writing – review & editing.

## Appendix

**Table A1:** Results for Arabic test scores of each subject (H = high-level; L = low-level).

**Subject no**.	**Test score (total 24)**	**Level group**	**Subject no**.	**Test score (total 24)**	**Level group**
1	13	L	21	19	H
2	18	H	22	18	H
3	20	H	23	23	H
4	9	L	24	22	H
5	10	L	25	15	L
6	20	H	26	14	L
7	15	L	27	9	L
8	16	L	28	19	L
9	15	H	29	15	H
10	17	H	30	23	H
11	22	L	31	5	L
12	21	H	32	7	L
13	17	L	33	19	L
14	12	L	34	2	L
15	22	H	35	19	H
16	18	L	36	20	H
17	23	H	37	19	L
18	13	L	38	18	L
19	23	H	39	20	H
20	17	H	40	22	H
